# SmYABBY1, a Light-Inducible Transcription Factor, Positively Regulates Anthocyanin Biosynthesis in Eggplant (*Solanum melongena* L.)

**DOI:** 10.3390/ijms27104347

**Published:** 2026-05-13

**Authors:** Suli Shi, Guozhi Yang, Zhanggen Gu, Qin Xue, Yang Liu, Lihua Ye

**Affiliations:** 1 Jiaxing Academy of Agricultural Sciences, Jiaxing 314000, China; 2 School of Agriculture and Biology, Shanghai Jiao Tong University, Shanghai 200240, China

**Keywords:** eggplant, SmYABBY1, anthocyanin, overexpression

## Abstract

Anthocyanin biosynthesis in eggplant (*Solanum melongena* L.) is highly light-dependent, and insufficient light severely impairs fruit coloration, which restricts the development of the eggplant industry. SmMYB75 is a key positive regulator of anthocyanin biosynthesis, but its regulatory partners remain unclear. In this study, seven *SmYABBY* genes were identified from the eggplant genome, all containing conserved zinc finger and YABBY domains. Expression analysis showed that *SmYABBY1* was predominantly expressed in fruit peel and significantly induced by light, with a peak at 4 h after light exposure. The yeast two-hybrid and bimolecular fluorescence complementation assays indicated that SmYABBY1 interacts with SmMYB75 and the light signaling regulator SmCOP1 in the nucleus. The heterologous overexpression of *SmYABBY1* in *Arabidopsis* enhanced anthocyanin accumulation and upregulated the expression of anthocyanin structural genes. Transient co-expression in tobacco leaves further demonstrated that SmYABBY1 synergistically enhances SmMYB75-mediated anthocyanin biosynthesis. The yeast one-hybrid and Dual-LUC assays revealed that SmYABBY1 does not directly bind to the promoters of *SmMYB75*, *SmDFR*, and *SmANS* but indirectly promotes their transcriptional activity. Our results illustrate that SmYABBY1 acts as a transcriptional co-activator, interacting with SmMYB75 to promote anthocyanin accumulation, while SmCOP1 is involved in this regulatory process. This study provides a molecular basis for improving eggplant coloration under suboptimal light conditions.

## 1. Introduction

Eggplant (*Solanum melongena* L.) is one of the most important solanaceous vegetables globally, with China contributing over 60% of the world’s total production (FAO, 2023). The purple pigmentation of eggplant peel is primarily attributed to anthocyanins, which not only determine fruit commercial quality but also offer health benefits to humans, including antioxidant, anti-inflammatory, and anti-cancer activities [1,2]. Moreover, anthocyanins serve as critical protective compounds for plants, shielding them from UV radiation, pathogen invasion, and oxidative damage [3,4]. However, anthocyanin biosynthesis in eggplant is highly light-dependent, and insufficient light caused by dense planting or protected cultivation leads to uneven fruit coloration and reduced nutritional quality, becoming a major constraint for the eggplant industry [5,6].

The molecular mechanism of anthocyanin biosynthesis has been well studied in plants. It is a branch of the flavonoid pathway, regulated by a series of structural genes (e.g., *CHS*, *CHI*, *F3H*, *DFR*, *ANS*) and transcription factors (TFs) [7,8,9]. The MYB-bHLH-WD40 (MBW) ternary complex is the core regulatory module, where MYB TFs directly bind to the promoters of structural genes to activate their expression [10,11,12]. In eggplant, SmMYB113 and SmMYB75 have been identified as positive regulators of anthocyanin biosynthesis *SmMYB75* overexpression lines show enhanced anthocyanin accumulation in all organs, even under low light [5,13]. However, the regulatory factors interacting with SmMYB75 to fine-tune anthocyanin biosynthesis remain largely unknown.

YABBY proteins are plant-specific TFs characterized by a conserved N-terminal zinc finger domain and a C-terminal YABBY domain. They are traditionally known to regulate leaf polarity establishment, flower development, and stress responses [14,15]. Recent studies have revealed their involvement in secondary metabolism: in *Arabidopsis*, AtYAB1 (FIL) binds to the *AtMYB75* promoter to promote anthocyanin accumulation [16]; in *Artemisia annua*, AaYABBY5 directly regulates artemisinin biosynthesis by targeting CYP71AV1 and DBR2 [17]. In eggplant, transcriptome analyses have implied that *SmYABBY* genes are responsive to light, suggesting their potential role in anthocyanin biosynthesis [18]. Additionally, a recent study reported that SmYABBY interacts with cold-responsive gene *SmICE1* to potentially regulate anthocyanin synthesis under low-temperature conditions [19], indicating the multi-signal integration function of SmYABBYs. However, the specific function and regulatory mechanism of SmYABBY transcription factors in light-induced anthocyanin accumulation remain unclear.

Light signal transduction plays a crucial role in regulating anthocyanin biosynthesis. The E3 ubiquitin ligase CONSTITUTIVELY PHOTOMORPHOGENIC1 (COP1) acts as a central negative regulator in light signaling [20,21]. In darkness, COP1 forms a complex with SPA proteins and mediates the ubiquitination and degradation of positive regulators such as HY5 and MYB TFs, thereby inhibiting anthocyanin synthesis [22,23]. Under light conditions, COP1 activity is suppressed, allowing the accumulation of positive regulators to activate anthocyanin biosynthesis [24,25]. Previous studies have shown that SmCOP1 interacts with SmMYB5 in eggplant, suggesting that SmMYB5 is regulated by the COP1-mediated ubiquitin-proteasome pathway (Supplementary Figure S1). However, whether SmCOP1 interacts with other transcription factors to modulate SmMYB75-mediated anthocyanin biosynthesis remains to be explored.

Anthocyanin biosynthesis is tightly regulated by the regulatory framework that links light signaling, COP1, and MBW complexes [26]. Light signals suppress the activity of COP1, thereby stabilizing the MBW complex and promoting the expression of anthocyanin biosynthetic structural genes. Meanwhile, numerous transcriptional coactivators and auxiliary regulatory proteins have been confirmed to modulate the activity of MBW complexes, thereby fine-tuning anthocyanin production [27].

In this study, we systematically identified the *SmYABBY* gene family in eggplant and focused on the functional analysis of *SmYABBY1*. Using a combination of eggplant expression analysis, *Arabidopsis* stable overexpression, tobacco transient expression, protein–protein interaction assays, and promoter activity detection, we demonstrated that SmYABBY1 physically interacts with SmMYB75 and SmCOP1 and acts as a transcriptional co-activator to indirectly enhance anthocyanin biosynthesis. Our findings reveal a novel light-responsive regulatory module in eggplant and provide important gene resources for improving fruit coloration under suboptimal light conditions.

## 2. Results

### 2.1. Identification and Phylogenetic Analysis of SmYABBY Genes

**Figure 1:**
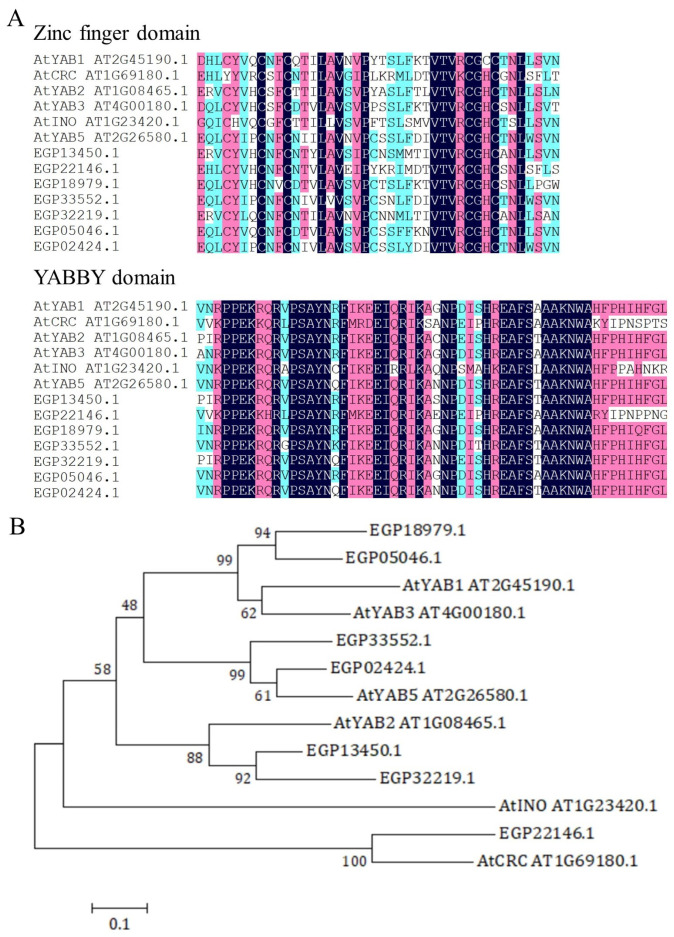
Amino acid sequence alignment analysis of SmYABBYs. (**A**) Results of multiple sequence alignment of amino acid sequences of SmYABBYs with six AtYABBY transcription factors of *Arabidopsis*. (**B**) Phylogenetic relationship of SmYABBYs proteins with YABBY transcription factors of *Arabidopsis*.

A total of 7 *SmYABBY* genes were identified from the eggplant genome. All predicted SmYABBY proteins contained N-terminal zinc finger domain and C-terminal YABBY domain, which are typical characteristics of the YABBY family (Figure 1A). Phylogenetic analysis showed that these *SmYABBYs* were clustered with *Arabidopsis* YABBYs into six subgroups: *SmYABBY1* (*EGP05046.1*) and *SmYABBY3* (*EGP18979.1*) grouped with *AtYAB1*/*AtYAB3*; *SmYABBY2* (*EGP13450.1*) and *SmYABBY2.1* (*EGP32219.1*) grouped with *AtYAB2*; *SmYABBY5* (*EGP33552.1*) and *SmYABBY5.1* (*EGP02424.1*) grouped with *AtYAB5*; and *SmCRC* (*EGP22146.1*) grouped with *AtCRC* (Figure 1B).

### 2.2. Gene Expression Pattern Analysis

**Figure 2:**
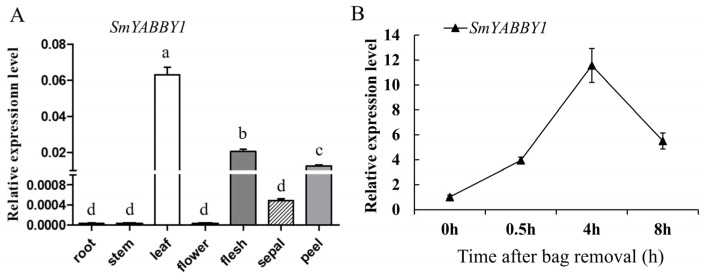
The expression patterns of the SmYABBY1 gene. (**A**) Expression level of SmYABBY1 in different tissues. (**B**) Expression level of SmYABBY1 in eggplant peels after removing the bag. The different lowercases indicate the statistical differences determined by Duncan’s New Multiple Range test (*p* ≤ 0.05) of variance (ANOVA) method.

qRT-PCR was performed to elucidate the expression patterns of *SmYABBY1* in seven different tissues and whether *SmYABBY1* expression is regulated by light. As shown in Figure 2A, *SmYABBY1* expression levels were significantly higher in leaves, flesh, and fruit peel compared to other tissues. After removing the bag for a period of time (0~8 h), the expression level of *SmYABBY1* increased successively, and the expression level at 4 h was about 11 times that at 0 h, followed by a gradual upward trend before decreasing (Figure 2B). These results indicated that SmYABBY1 is a light-inducible gene and may participate in light-dependent anthocyanin biosynthesis in eggplant peel.

### 2.3. SmYABBY1 Promotes Anthocyanin Biosynthesis Accumulation in Arabidopsis

**Figure 3:**
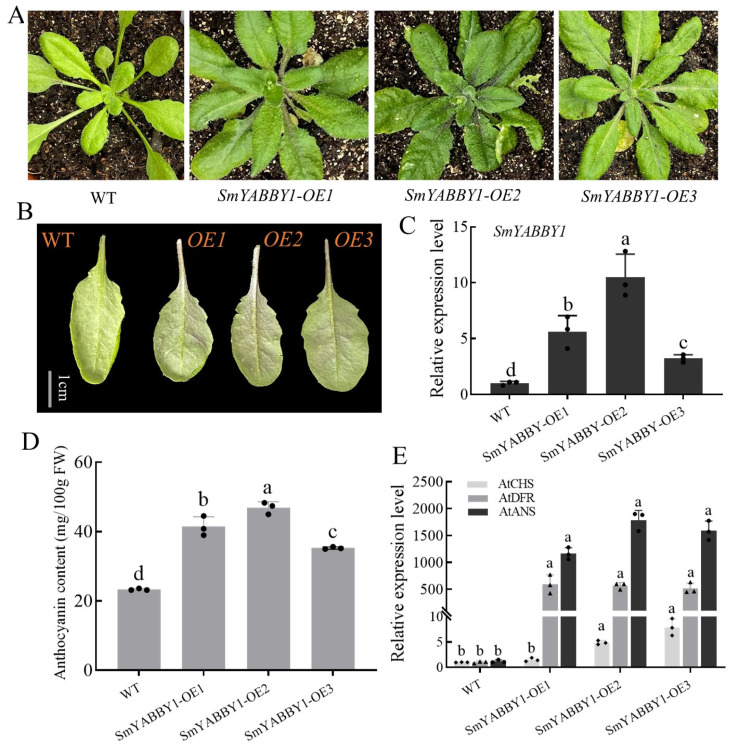
Functional validation analysis of *SmYABBY1* gene in *Arabidopsis thaliana*. (**A**) Comparative phenotypic analysis of *SmYABBY1* transgenic lines and wild-type *Arabidopsis* plants. (**B**) Comparative phenotypic analysis of *SmYABBY1* transgenic lines and wild-type *Arabidopsis* leaves abaxially. (**C**) qRT-PCR detection of *SmYABBY1* gene expression in transgenic *Arabidopsis*. (**D**) Anthocyanin content in leaves of *SmYABBY1* transgenic lines and wild-type lines. (**E**) Expression of structural anthocyanin synthesis genes in transgenic *Arabidopsis* by qRT-PCR. Three biological replicates were used for all samples, and the error line represents the standard error between the three replicates. The different lowercases indicate the statistical differences determined by Duncan’s New Multiple Range test (*p* < 0.05) of variance (ANOVA) method.

To investigate the function of *SmYABBY1*, we generated transgenic *Arabidopsis* lines overexpressing *SmYABBY1*. Compared with wild-type plants, the leaves of transgenic lines exhibited obvious purple pigmentation (Figure 3A,B). qRT-PCR showed that *SmYABBY1* expression was significantly higher in transgenic lines than in the wild type (Figure 3C). The anthocyanin content in transgenic lines was ~2-fold higher than in the wild type (Figure 3D), and the expression levels of anthocyanin structural genes *AtCHS*, *AtDFR*, and *AtANS* were significantly upregulated (Figure 3E). These results demonstrated that *SmYABBY1* enhances anthocyanin accumulation in a heterologous system.

### 2.4. SmYABBY1 Enhances SmMYB75-Mediated Anthocyanin Biosynthesis in Tobacco

**Figure 4:**
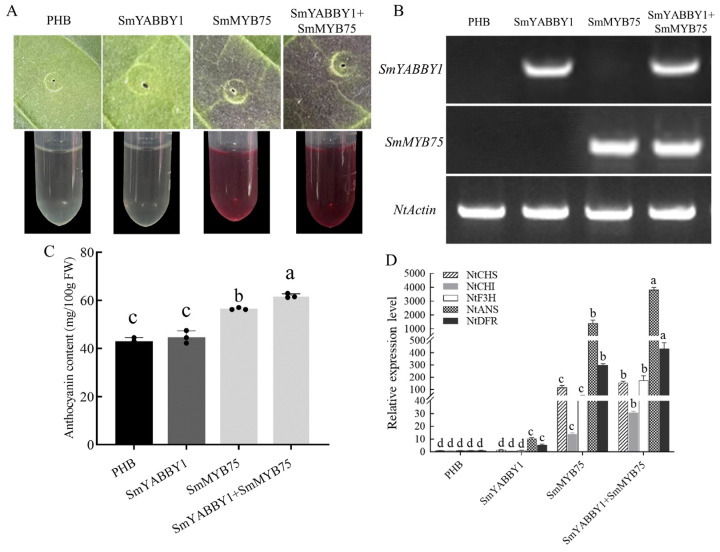
Transient overexpression assay of *SmMYB75* and *SmYABBY1*. (**A**) Phenotypes of tobacco leaves 4 days after injection of SmMYB75 and SmYABBY1. (**B**) RT-PCR assays for expression levels of *SmMYB75* and *SmYABBY1* in injected tobacco leaf sites. (**C**) Anthocyanin content. (**D**) qRT-PCR analysis of tobacco anthocyanin biosynthesis genes: *NtCHS* (*AF311783*), *NtCHI* (*AB213651*), *NtF3H* (*AB289450*), *NtDFR* (*EF421429*) and *NtANS* (*AB723683*). The infiltrated empty vector PHB was used as a control. Error bars indicate the standard error of three biological replicates. Different lowercase letters indicate statistical differences determined by Duncan’s new multiple range test (*p* < 0.05) by the method of variance (ANOVA).

Transient expression in tobacco leaves was used to verify the genetic interaction between SmYABBY1 and SmMYB75. The expression of *SmMYB75* alone significantly induced anthocyanin accumulation, whereas the co-expression of *SmYABBY1* and *SmMYB75* further deepened pigmentation and increased anthocyanin content compared with *SmMYB75* alone (Figure 4A,C). The transcription level of SmMYB75 was similar in single and co-expression tissues (Figure 4B). The expression levels of anthocyanin structural genes of *NtCHS*, *NtCHI*, *NtF3H*, *NtDFR*, and *NtANS* was significantly higher in co-expression lines than in *SmMYB75* alone lines (Figure 4D). These results indicated that SmYABBY1 acts synergistically with SmMYB75 to promote anthocyanin biosynthesis.

### 2.5. Interaction Between SmYABBYs and SmMYB75

**Figure 5:**
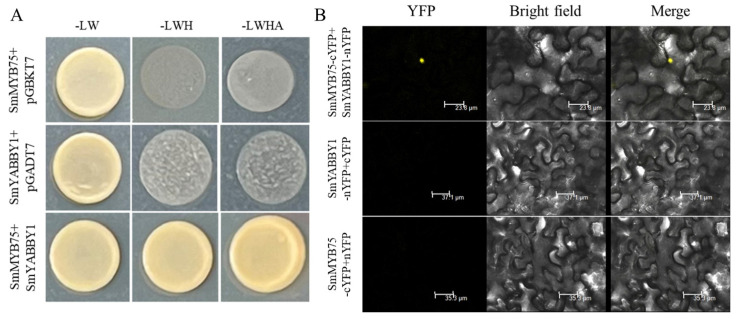
Interaction between SmYABBY1 and SmMYB75. (**A**) The interaction between SmYABBY1 and SmMYB75 was analyzed by the yeast two-hybrid (Y2H) assay. (**B**) Bimolecular Fluorescence Complementation (BiFC) assay confirmed the interaction between SmYABBY1 and SmMYB75 in planta. YFP: YFP fluorescence channel; Bright field: Bright field channel; Merge: Merged channel.

To explore the regulatory mechanism, we performed protein–protein interaction assays. Y2H assays showed that yeast cells co-transformed with SmYABBY1-pGBKT7 and SmMYB75-pGADT7 could grow on SD-TLHA medium, indicating an in vitro interaction between SmYABBY1 and SmMYB7 (Figure 5A). BiFC assays showed that the co-expression of SmYABBY1-nYFP and SmMYB75-cYFP produced strong YFP fluorescence in the nucleus of tobacco cells, revealing in planta interaction and nuclear colocalization (Figure 5B). These results support the functional interaction between SmYABBY1 and SmMYB75.

### 2.6. SmYABBY1 Interacts with SmCOP1

**Figure 6:**
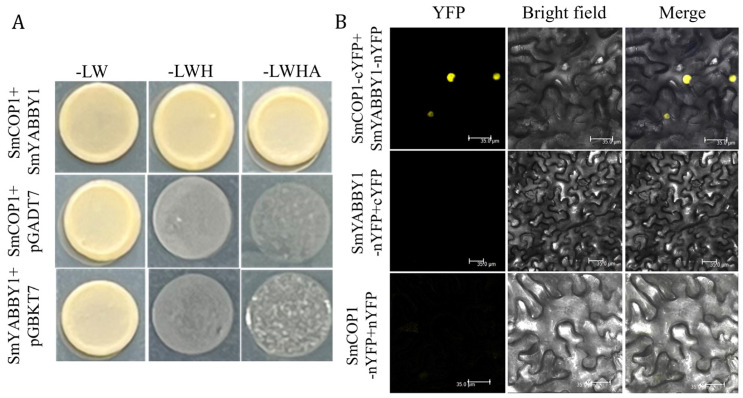
Interaction between SmYABBY1 and SmCOP1. (**A**) The interaction between SmYABBY1 and SmCOP1 was analyzed by the yeast two-hybrid (Y2H) assay. (**B**) Bimolecular Fluorescence Complementation (BiFC) assay confirmed the interaction between SmYABBY1 and SmCOP1 in planta. YFP: YFP fluorescence channel; Bright field: Bright field channel; Merge: Merged channel.

Considering that SmYABBY1 is light-inducible, we further tested its interaction with SmCOP1. Y2H assays showed that yeast cells co-transformed with SmYABBY1-pGADT7 + SmCOP1-pGBKT7 could grow on SD-LWHA medium, while the negative controls could not do so, indicating that SmYABBY1 interacts with SmCOP1 (Figure 6A). BiFC assays confirmed that SmYABBY1-nYFP co-localized with SmCOP1-cYFP in the nucleus, producing YFP fluorescence (Figure 6B). These results suggested that SmYABBY1 is involved in light signaling through interaction with SmCOP1.

### 2.7. SmYABBY1 Indirectly Activates the Expression of SmMYB75, SmDFR, and SmANS

**Figure 7:**
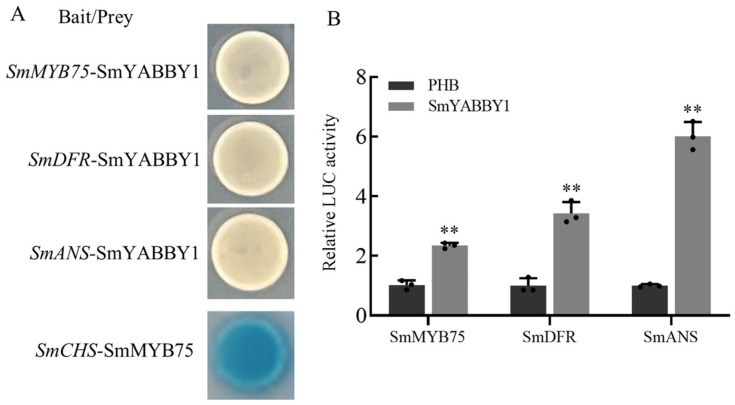
The yeast one-hybrid and Dual-LUC experiments verified that SmYABBY1 activates the expression of *SmMYB75*, *SmDFR* and *SmANS*. (**A**) Specific binding of SmYABBY1 to the promoters of the three genes was observed by a one-hybrid system. (**B**) Dual-LUC experiment. *SmYABBY1-YFP* was used as effector and PHB vector as negative control. *SmMYB75*, *SmDFR*, and *SmANS* promoters were fused to the pGreenII0800-LUC vector as reporter genes, respectively. The binding ability of SmYABBY1 to the promoter is expressed as the ratio of LUC to REN. Error bars indicate the standard error of three biological replicates. Asterisks indicate statistically significant differences (**, *p* < 0.01).

To clarify the transcriptional regulatory mechanism, the yeast one-hybrid (Y1H) and Dual-LUC assays were performed. Y1H assays showed no blue coloration in yeast cells co-transformed with SmYABBY1-pB42AD and SmMYB75-LacZ, SmDFR-LacZ, or SmANS-LacZ, indicating no direct binding (Figure 7A). Dual-LUC assays showed that overexpression of SmYABBY1 increased the activity of *SmMYB75*, *SmDFR*, and *SmANS* promoters by 2.34-, 3.42-, and 6.01-fold, respectively, compared to the PHB control (Figure 7B). These results demonstrated that SmYABBY1 does not directly bind target promoters but enhances their transcription through an indirect regulatory mechanism.

## 3. Discussion

YABBY transcription factors are unique to seed plants, traditionally recognized for their roles in leaf polarity establishment, flower and fruit development, and abiotic stress responses [28,29]. Previous studies have reported that YABBY proteins participate in the regulatory network of flavonoid biosynthesis, including anthocyanins and flavones. For instance, YAB1 (FIL) in *Arabidopsis thaliana* can bind to the promoter of *AtMYB75* and activate its expression, leading to anthocyanin accumulation [16]. Additionally, in the medicinal plant *Artemisia annua*, AaYABBY5 directly targets cytochrome P450 monooxygenase (CYP71AV1) and double bond reductase 2 (DBR2), which are involved in artemisinin biosynthesis, thereby positively regulating artemisinin production [30]. In eggplant, transcriptomic analyses have previously suggested that *SmYABBY* genes are light-responsive [18], and SmYABBYs may interact with cold-responsive SmICE1 to modulate anthocyanin synthesis under low temperatures [19], implying their potential integration of multiple environmental signals in metabolic regulation. Our study builds on these findings by systematically identifying SmYABBY family members in eggplant and elucidating the specific role of SmYABBY1 in anthocyanin biosynthesis through interactions with SmMYB75 and SmCOP1.

Genome-wide identification revealed seven *SmYABBY* genes in eggplant, all harboring the conserved N-terminal zinc finger domain and C-terminal YABBY domain characteristic of this family (Figure 1A) [31,32]. Phylogenetic analysis clustered these genes with *Arabidopsis* YABBY subgroups, suggesting functional conservation and divergence (Figure 1B). SmYABBY1 and SmYABBY3 grouped with AtYAB1/AtYAB3, which are involved in anthocyanin [28]. This phylogenetic placement aligns with our subsequent functional validation, highlighting the predictive value of evolutionary analysis for gene function.

Consistent with previous studies, the regulatory network connecting light, COP1, and MBW complexes serves as a conserved and essential mechanism in light-induced anthocyanin biosynthesis [26]. Moreover, transcriptional coactivators and auxiliary regulatory proteins often participate in this pathway by interacting with MBW complexes to enhance their stability and transcriptional activation capacity [27].

Expression pattern analysis provided critical insights into SmYABBY1’s biological role. *SmYABBY1* was predominantly expressed in fruits, particularly in the peel (Figure 2A), which is the major site of anthocyanin accumulation in eggplant [33]. Notably, *SmYABBY1* expression was dynamically induced by light: after bag removal, its transcript levels increased significantly, peaking at 4 h (Figure 2B). This light responsiveness, combined with the presence of light-responsive cis-acting elements in its promoter (Supplementary Figure S2), confirms SmYABBY1 as a light-regulated gene, further linking it to the light-dependent anthocyanin synthesis pathway addressed in our study.

Functional validation in tobacco and *Arabidopsis* heterologous systems directly demonstrated SmYABBY1’s role in promoting anthocyanin biosynthesis. The heterologous overexpression of *SmYABBY1* in *Arabidopsis* resulted in purple leaf phenotypes, significantly higher anthocyanin content, and the upregulated expression of key structural genes (*AtCHS*, *AtDFR*, *AtANS*) compared to wild-type plants (Figure 3). This confirms that *SmYABBY1* can independently drive anthocyanin accumulation across plant species, highlighting its conserved function in regulating the flavonoid pathway. Although native functional validation such as gene silencing or genome editing in eggplant was not performed in this study, tobacco and *Arabidopsis* are widely used and reliable heterologous systems for transcription factor functional analysis. Further, transient co-expression assays in tobacco revealed a synergistic effect between SmYABBY1 and SmMYB75: co-expression led to more intense pigmentation, higher anthocyanin content, and stronger activation of structural genes (*NtCHS*, *NtCHI*, *NtF3H*, *NtDFR*, *NtANS*) than SmMYB75 alone (Figure 4). This finding indicates that SmYABBY1 acts as a co-activator to enhance SmMYB75-mediated anthocyanin biosynthesis, a regulatory mode analogous to the interaction between PpERF24/PpERF96 and the PpMYB114-PpbHLH3 complex in pear [34], where protein–protein interaction amplifies metabolic signals.

Protein interaction assays revealed that SmYABBY1 interacts and colocalizes with SmMYB75 in the nucleus, supporting their functional interaction (Figure 5). It should be noted that these assays indicate in planta interaction and nuclear localization rather than definitive direct physical binding. To clarify how this interaction regulates downstream genes, the yeast one-hybrid and Dual-LUC assays were performed. The results showed that SmYABBY1 does not directly bind to the promoters of *SmMYB75*, *SmDFR*, or *SmANS* (Figure 7A) but significantly enhances their promoter activity (Figure 7B). This difference can be explained by the lack of SmMYB75 and MBW complex components in the yeast one-hybrid system, which cannot fully represent the native transcriptional environment in plant cells. We propose that SmYABBY1 modulates transcription as a transcriptional co-activator likely by interacting with SmMYB75 to strengthen its transcriptional activation capacity, a common mechanism in anthocyanin synthesis pathways.

**Figure 8:**
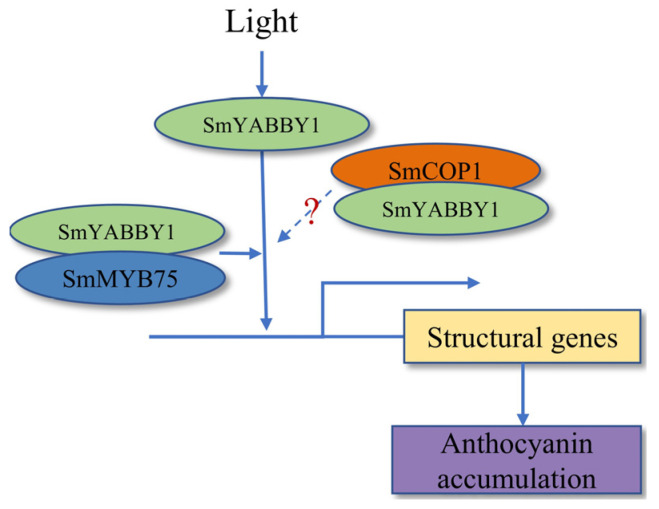
A putative model for the role of SmYABBY1 and SmMYB75 in anthocyanin accumulation in eggplant.

Notably, we identified SmCOP1 as a novel interacting partner of SmYABBY1 (Figure 6). As a core negative regulator of light signaling, SmCOP1 mediates the ubiquitination and degradation of positive regulators such as MYB TFs and HY5 [35,36]. Their interaction implies that SmYABBY1’s stability or activity is regulated by the COP1-mediated ubiquitin-proteasome pathway, as reported for AtMYB75/PAP1 in *Arabidopsis* [37], integrating SmYABBY1 into the COP1-centered light signaling network. Collectively, these results form a novel light-regulated module for SmYABBY1-mediated anthocyanin biosynthesis in eggplant: light induces SmYABBY1 expression, and SmYABBY1 interacts with SmMYB75 to indirectly promote anthocyanin structural genes; meanwhile, SmCOP1 interacts with SmYABBY1 to fine-tune this process (Figure 8). This model identifies SmYABBY1 as a key node connecting light signaling to anthocyanin metabolic output, explaining the light sensitivity of eggplant anthocyanin biosynthesis and expanding our understanding of the regulatory network controlling anthocyanin biosynthesis in solanaceous vegetables.

Several questions remain to be addressed in future studies. First, the specific domains mediating the SmYABBY1-SmMYB75 and SmYABBY1-SmCOP1 interactions need to be identified to clarify the molecular basis of these interactions. Second, the ubiquitination sites on SmYABBY1 targeted by SmCOP1 should be characterized to confirm the regulatory mechanism of SmCOP1. Third, the potential involvement of bHLH and WD40 proteins (forming the MBW complex) in the SmYABBY1-SmMYB75 module warrants investigation, as bHLH proteins often stabilize MYB interactions and enhance transcriptional activity [38,39].

This study systematically characterizes SmYABBY1 as a light-inducible regulator of anthocyanin biosynthesis in eggplant. Through interactions with SmMYB75 and SmCOP1, SmYABBY1 integrates light signals to enhance anthocyanin synthesis, revealing a new regulatory module that connects light signaling and anthocyanin metabolism. These findings not only advance our understanding of *YABBY* gene function but also offer practical targets for improving eggplant fruit quality under suboptimal light conditions.

## 4. Materials and Methods

### 4.1. Plant Materials and Growth Conditions

The eggplant (*Solanum melongena* L.) cultivar ‘Lanshan Hexian’ (purple peel with high anthocyanin content) was transplanted to the Horticultural Farm of Jiaxing Academy of Agricultural Sciences, Jiaxing, China. Root, stem, leaf, flower, sepal, flesh, and pericarp samples were collected from the same individual plants to analyze tissue-specific gene expression. At full bloom, the sepals of eggplant flowers were covered with opaque paper bags. After 14 days, the developing eggplant fruits were harvested and subjected to continuous light treatment in the laboratory. Pericarp samples were collected at 0 h, 0.5 h, 4 h, and 8 h post-treatment, with three independent eggplant fruits selected as biological replicates. All freshly collected plant materials were immediately frozen in liquid nitrogen and stored at −80 °C until further use.

Tobacco (*Nicotiana tabacum* L. and *Nicotiana benthamiana*) and *Arabidopsis thaliana* (*Columbia-0* ecotype) were also used in this study. All these plants were grown in a growth chamber under conditions of 25 ± 1 °C and a 16 h light/8 h dark photoperiod.

### 4.2. Isolation and Phylogenetic Evolution Analysis of Eggplant SmYABBY Genes

*Arabidopsis* YABBY protein sequences (AtYAB1-AtYAB5, AtCRC) were retrieved from TAIR (https://www.arabidopsis.org/, accessed on 1 March 2026). BLASTp searches were performed against the eggplant genome database (https://db.cngb.org/search/project/CNP0000734/, accessed on 1 March 2026) to identify homologous SmYABBY genes. The conserved YABBY domain was verified using the CDD database (https://www.ncbi.nlm.nih.gov/Structure/cdd/cdd.shtml, accessed on 1 March 2026). Multiple sequence alignment was conducted with ClustalW, and a phylogenetic tree was constructed using MEGA11 (Mega Limited, Auckland, New Zealand) with the neighbor-joining method (1000 bootstrap replicates).

### 4.3. qRT-PCR Analysis

qRT-PCR was performed to analyze the tissue expression specificity of SmYABBYs and the expression levels of anthocyanin biosynthesis structural genes in all plant materials in this study. A total of 1 μg RNA was used for the synthesis of cDNA. qRT-PCR was performed with LightCycler 96 (Roche, Basel, Switzerland) utilizing SYBR Premix Ex Taq II Kit (Takara, Kyoto, Japan). The specific PCR procedure was described in previous research [18]. Each sample was set with three biological replicates and three technical replicates. The *Actin* gene (*GU984779.1*) was used as a constitutive control. The relative expression levels were analyzed using the 2^−ΔΔCt^ method [40]. All gene-specific primers used in this study are listed in Supplementary Table S1.

### 4.4. Determination of Anthocyanin Content

The content of anthocyanin in 0.5 g callus of eggplant was measured by the pH differential spectrophotometry approach used in previous studies [5]. The calculation formula of anthocyanin content is as follows: TA = A × MW × 5 × 100 × V/e; here, TA represents the total anthocyanin content (mg/100 g), the value of V is the final volume (ml), and A = [A510 nm (pH 1.0) − A700 nm (pH 1.0)] − [A510 nm (pH 4.5) − A700 nm (pH 4.5)]. Three biological replicates were performed on each sample.

### 4.5. Yeast Two-Hybrid (Y2H) Assay

The full-length CDS of SmYABBYs genes was cloned into the pGBKT7 vector (bait), and SmMYB75 CDS was cloned into the pGADT7 vector (prey). Bait and prey vectors were co-transformed into yeast strain AH109. Transformed yeasts were plated on SD/-Trp-Leu (TL) medium for selection, and positive clones were transferred to SD/-Trp-Leu-His-Ade (TLHA) medium to verify interactions.

### 4.6. Bimolecular Fluorescence Complementation (BiFC) Assay

SmYABBYs CDS (without stop codon) was cloned into pXY106 (nYFP), and SmMYB75 or SmCOP1 CDS (without stop codon) was cloned into pXY104 (cYFP). The recombinant vectors were co-transformed into *Agrobacterium tumefaciens* GV3101, which was then infiltrated into *N. benthamiana* leaves. YFP fluorescence was observed 48 h post-infiltration using a laser confocal microscope (Leica TCS SP8, Leica Microsystems, Wetzlar, Germany).

### 4.7. Heterologous Overexpression of SmYABBYs in Arabidopsis

SmYABBYs CDS was cloned into the PHB-YFP vector and transformed into A. tumefaciens *GV3101*. *Arabidopsis* transformation was performed using the floral dip method. Transgenic plants were selected on MS medium containing 50 mg·L^−1^ hygromycin, and T3 homozygous lines were identified by PCR and qRT-PCR. Anthocyanin content in leaves was determined using the pH differential method, and the expression of anthocyanin biosynthesis-related genes was analyzed by qRT-PCR.

### 4.8. Transient Co-Expression in Tobacco Leaves

SmYABBY1 and SmMYB75 CDS were cloned into the PHB-YFP vector. *A. tumefaciens* strains containing PHB-SmYABBY1-YFP, PHB-SmMYB75-YFP, or empty PHB-YFP were infiltrated into *N. tabacum* leaves. Four days post-infiltration, leaf phenotypes were observed, anthocyanin content was measured, and the expression of *NtCHS*, *NtCHI*, *NtF3H*, *NtDFR*, and *NtANS* was analyzed by qRT-PCR.

### 4.9. Vector Construction and Promoters

For heterologous expression in *Arabidopsis* stable transformation, tobacco transient assays, and other heterologous systems used in this study, the full-length coding sequences of *SmYABBY1* and *SmMYB75* were cloned into the PHB-YFP vector.

The *CaMV 35S* promoter was used to drive the expression of *SmYABBY1* and *SmMYB75* in all these heterologous systems.

### 4.10. Yeast One-Hybrid (Y1H) and Dual-Luciferase (Dual-LUC) Assays

For Y1H, the promoters of *SmMYB75*, *SmDFR*, and *SmANS* were cloned into *placZ2u* (reporter), and SmYABBY1 CDS was cloned into *pB42AD* (effector). Vectors were co-transformed into yeast strain EGY48a, and interactions were verified on SD/-Ura/-Trp medium supplemented with X-gal. For Dual-LUC, the promoters of *SmMYB75*, *SmDFR*, and *SmANS* were cloned into *pGreenII0800-LUC* (reporter), and SmYABBY1-YFP was used as the effector. The reporter and effector vectors were co-transformed into *N. benthamiana* leaves. LUC and REN luciferase activities were measured using the Dual-Luciferase Reporter Assay System (Promega, Madison, WI, USA), and the LUC/REN ratio was calculated to evaluate promoter activation.

### 4.11. Statistical Analysis

All experiments were performed with three biological replicates. Data were analyzed using SPSS 26.0 (IBM, Chicago, IL, USA) software, and significant differences were determined by one-way ANOVA, followed by Duncan’s multiple range test (*p* < 0.05) or *t*-test (*p* < 0.01).

## 5. Conclusions

In summary, this study identified a light-inducible YABBY transcription factor, SmYABBY1, from eggplant. Expression patterns showed that *SmYABBY1* was highly expressed in colored peels and strongly induced by light. Functional assays in tobacco and *Arabidopsis* demonstrated that *SmYABBY1* significantly promotes anthocyanin accumulation in planta. Mechanistically, SmYABBY1 does not directly bind to the promoters of anthocyanin biosynthetic genes but physically interacts with SmMYB75 and SmCOP1 to form a regulatory module. SmYABBY1 acts as a transcriptional co-activator to enhance the transcriptional activity of SmMYB75, thereby indirectly promoting the expression of downstream structural genes and anthocyanin biosynthesis. Meanwhile, the function of *SmYABBY1* is modulated by light signaling and the photo-responsive factor SmCOP1. These findings reveal a novel regulatory mechanism of YABBY proteins in light-mediated anthocyanin metabolism and provide a valuable target for the molecular breeding of eggplant fruit color.

## Data Availability

The original contributions presented in this study are included in the article/Supplementary Materials. Further inquiries can be directed to the corresponding authors.

## Supplementary Materials

The following supporting information can be downloaded at https://www.mdpi.com/article/10.3390/ijms27104347/s1.

## References

[1] He J., Giusti M.M. (2010). Anthocyanins: Natural Colorants with Health-Promoting Properties. Annu. Rev. Food Sci. Technol..

[2] Li S., He Y., Li L., Li D., Chen H. (2022). New insights on the regulation of anthocyanin biosynthesis in purple Solanaceous fruit vegetables. Sci. Hortic..

[3] Winkel-Shirley B. (2002). Biosynthesis of flavonoids and effects of stress. Curr. Opin. Plant Biol..

[4] Stintzing F.C., Carle R. (2004). Functional properties of anthocyanins and betalains in plants, food, and in human nutrition. Trends Food Sci. Technol..

[5] Shi S., Liu Y., He Y., Li L., Li D., Chen H. (2021). R2R3-MYB transcription factor SmMYB75 promotes anthocyanin biosynthesis in eggplant (*Solanum melongena* L.). Sci. Hortic..

[6] Jiang M., Ren L., Lian H., Liu Y., Chen H. (2016). Novel insight into the mechanism underlying light-controlled anthocyanin accumulation in eggplant (*Solanum melongena* L.). Plant Sci..

[7] Tanaka Y., Ohmiya A. (2008). Seeing is believing: Engineering anthocyanin and carotenoid biosynthetic pathways. Curr. Opin. Biotechnol..

[8] Shi S., Li D., Li S., Zhao N., Liao J., Ge H., Liu Y., Chen H. (2024). Genome-Wide Analysis of R2R3-MYB Genes and Functional Characterization of SmMYB75 in Eggplant Fruit Implications for Crop Improvement and Nutritional Enhancement. Int. J. Mol. Sci..

[9] Li S., Dong Y., Li D., Shi S., Zhao N., Liao J., Liu Y., Chen H. (2024). Eggplant transcription factor SmMYB5 integrates jasmonate and light signaling during anthocyanin biosynthesis. Plant Physiol..

[10] Koes R., Verweij W., Quattrocchio F. (2005). Flavonoids: A colorful model for the regulation and evolution of biochemical pathways. Trends Plant Sci..

[11] Lepiniec L., Debeaujon I., Routaboul J.M., Baudry A., Pourcel L., Nesi N., Caboche M. (2006). Genetics and biochemistry of seed flavonoids. Annu. Rev. Plant Biol..

[12] Bulanov A.N., Andreeva E.A., Tsvetkova N.V., Zykin P.A. (2025). Regulation of Flavonoid Biosynthesis by the MYB-bHLH-WDR (MBW) Complex in Plants and Its Specific Features in Cereals. Int. J. Mol. Sci..

[13] Yang G., Li L., Wei M., Li J., Yang F. (2022). SmMYB113 is a key transcription factor responsible for compositional variation of anthocyanin and color diversity among eggplant peels. Front. Plant Sci..

[14] Yang C., Ma Y., Li J. (2016). The rice YABBY4 gene regulates plant growth and development through modulating the gibberellin pathway. J. Exp. Bot..

[15] Hou H., Wu P., Gao L., Zhang C., Hou X. (2019). Characterization and expression profile analysis of YABBY family genes in Pak-choi (*Brassica rapa* ssp. *chinensis*) under abiotic stresses and hormone treatments. Plant Growth Regul..

[16] Boter M., Golz J.F., Gimenez-Ibanez S., Fernandez-Barbero G., Franco-Zorrilla J.M., Solano R. (2015). FILAMENTOUS FLOWER Is a Direct Target of JAZ3 and Modulates Responses to Jasmonate. Plant Cell.

[17] Kayani S.-I., Shen Q., Ma Y., Fu X., Xie L., Zhong Y., Tiantian C., Pan Q., Li L., Rahman S.U. (2019). The YABBY Family Transcription Factor AaYABBY5 Directly Targets Cytochrome P450 Monooxygenase (CYP71AV1) and Double-Bond Reductase 2 (DBR2) Involved in Artemisinin Biosynthesis in *Artemisia annua*. Front. Plant Sci..

[18] Gu K.-D., Wang C.-K., Hu D.-G., Hao Y.-J. (2019). How do anthocyanins paint our horticultural products?. Sci. Hortic..

[19] Lu Z., Yang L., Qunxiang C., Huoying C. (2022). cloning and functional analysis of cold response gene SmICE1 in eggplant. J. Nanjing Agric. Univ..

[20] Li Y.Y., Mao K., Zhao C., Zhao X.Y., Zhang H.L., Shu H.R., Hao Y.J. (2012). MdCOP1 Ubiquitin E3 Ligases Interact with MdMYB1 to Regulate Light-Induced Anthocyanin Biosynthesis and Red Fruit Coloration in Apple. Plant Physiol..

[21] Maier A., Schrader A., Kokkelink L., Falke C., Welter B., Iniesto E., Rubio V., Uhrig J.F., Hulskamp M., Hoecker U. (2013). Light and the E3 ubiquitin ligase COP1/SPA control the protein stability of the MYB transcription factors PAP1 and PAP2 involved in anthocyanin accumulation in *Arabidopsis*. Plant J..

[22] Liu B., Zuo Z., Liu H., Liu X., Lin C. (2011). *Arabidopsis* cryptochrome 1 interacts with SPA1 to suppress COP1 activity in response to blue light. Genes Dev..

[23] Ma A., Wang D., Lu H., Wang H., Qin Y., Hu G., Zhao J. (2021). LcCOP1 and LcHY5 control the suppression and induction of anthocyanin accumulation in bagging and debagging litchi fruit pericarp. Sci. Hortic..

[24] Zhang Q., Lin L., Fang F., Cui B., Zhu C., Luo S., Yin R. (2022). Dissecting the functions of COP1 in the UVR8 pathway with a COP1 variant in *Arabidopsis*. Plant J..

[25] Xu D., Jiang Y., Li J., Lin F., Holm M., Deng X.W. (2016). BBX21, an *Arabidopsis* B-box protein, directly activates HY5 and is targeted by COP1 for 26S proteasome-mediated degradation. Proc. Natl. Acad. Sci. USA.

[26] Manickam G.P., Arul L., Sathiyamurthy V.A., Vijayalakshmi D., Kumar K.K. (2025). Anthocyanin pathway in eggplant: Genetic regula-tion and future directions for metabolic engineering. Mol. Biol. Rep..

[27] Li Y., Shan X., Gao R., Han T., Zhang J., Wang Y., Kimani S.K., Wang L., Gao X. (2020). MYB repressors and MBW activation complex collaborate to fine-tune flower coloration in *Freesia hybrida*. Commun. Biol..

[28] Yang T., He Y., Niu S., Zhang Y. (2022). A YABBY Gene CRABS CLAW a (CRCa) Negatively Regulates Flower and Fruit Sizes in Tomato. Plant Sci..

[29] Zeng D., Si C., Teixeira da Silva J.A., Dai G., Duan J., He C. (2023). Characterization of YABBY Genes in Dendrobium officinale Reveals Their Potential Roles in Flower Development. Protoplasma.

[30] Kayani S.I., Shen Q., Rahman S.U., Fu X., Li Y., Wang C., Hassani D., Tang K. (2021). Transcriptional Regulation of Flavonoid Biosynthesis in *Artemisia annua* by AaYABBY5. Hortic. Res..

[31] Xia J., Wang D., Peng Y., Wang W., Wang Q., Xu Y., Li T., Zhang K., Li J., Xu X. (2021). Genome-Wide Analysis of the YABBY Transcription Factor Family in Rapeseed (*Brassica napus* L.). Genes.

[32] Yin S., Li S., Gao Y., Bartholomew E.S., Wang R., Yang H., Liu C., Chen X., Wang Y., Liu X. (2022). Genome-Wide Identification of YABBY Gene Family in Cucurbitaceae and Expression Analysis in Cucumber (*Cucumis sativus* L.). Genes.

[33] Li Y., Xing M., Yang Q., Wang Y., Jiang J., Zhao Y., Zhao X., Shen A., Feng Y., Zhao X. (2023). SmCIP7, a COP1 Interactive Protein, Positively Regulates Anthocyanin Accumulation and Fruit Size in Eggplant. Int. J. Biol. Macromol..

[34] Ni J., Bai S., Zhao Y., Qian M., Tao R., Yin L., Gao L., Teng Y. (2019). Ethylene Response Factors Pp4ERF24 and Pp12ERF96 Regulate Blue Light-Induced Anthocyanin Biosynthesis in ‘Red Zaosu’ Pear Fruits by Interacting with MYB114. Plant Mol. Biol..

[35] Li Z., Liu W., Chen Q., Zhang S., Mei Z., Yu L., Wang C., Mao Z., Chen Z., Chen X. (2023). Mdm-miR858 Targets MdMYB9 and MdMYBPA1 to Participate in Anthocyanin Biosynthesis in Red-Fleshed Apple. Plant J..

[36] Lasmar dos Reis G., Zsögön A., Chalfun-Junior A., Peres L.E.P., Benedito V.A. (2026). Beyond HY5: COP1 Posttranslational Control of Anthocyanin Biosynthesis Proteins in Horticultural Crops. Plants.

[37] Zhou L.L., Zeng H.N., Shi M.Z., Xie D.Y. (2008). Development of Tobacco Callus Cultures Overexpressing *Arabidopsis* PAP1/MYB75 Transcription Factor and Characterization of Anthocyanin Biosynthesis. Planta.

[38] Ramsay N.A., Glover B.J. (2005). MYB-bHLH-WD40 Protein Complex and the Evolution of Cellular Diversity. Trends Plant Sci..

[39] Schaart J.G., Hekkert B.T., Vandenbussche M., Maliepaard C.A., Bovy A.G. (2013). Identification and Characterization of MYB-bHLH-WD40 Regulatory Complexes Controlling Proanthocyanidin Biosynthesis in Strawberry (*Fragaria* × *ananassa*) Fruits. New Phytol..

[40] Chen J., Jiang S., Yang G., Li L., Li J., Yang F. (2024). The MYB transcription factor SmMYB113 directly regulates ethylene-dependent flower abscission in eggplant. Plant Physiol. Biochem..

