# Supplementary material for: SmYABBY1, a Light-Inducible Transcription Factor, Positively Regulates Anthocyanin Biosynthesis in Eggplant (Solanum melongena L.)
Source: Int J Mol Sci. 2026 May 13;27(10):4347. doi: 10.3390/ijms27104347 (PMC13207839; doi:10.3390/ijms27104347)
Supplement: Supplementary file 1 [file ijms-27-04347-s001.zip › supplementary figures.pdf]

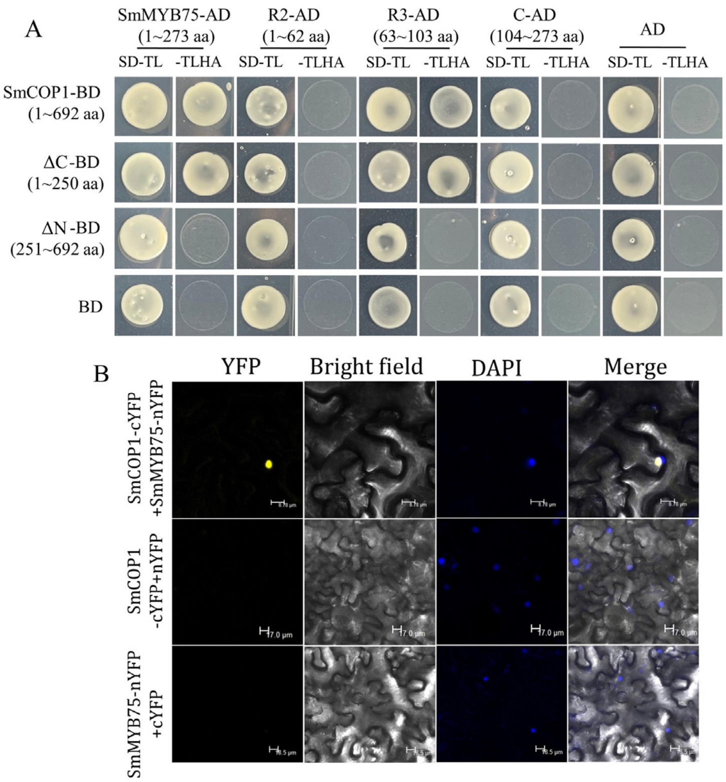

Supplementary Figure S1. The interaction between SmMYB75 and SmCOP1.

A. Analysis of the interaction between SmMYB75 and SmCOP1 by yeast two-hybrid assays.

B. SmMYB75 interacted specifically with SmCOP1 in BiFC assays using tobacco leaf epidermis.

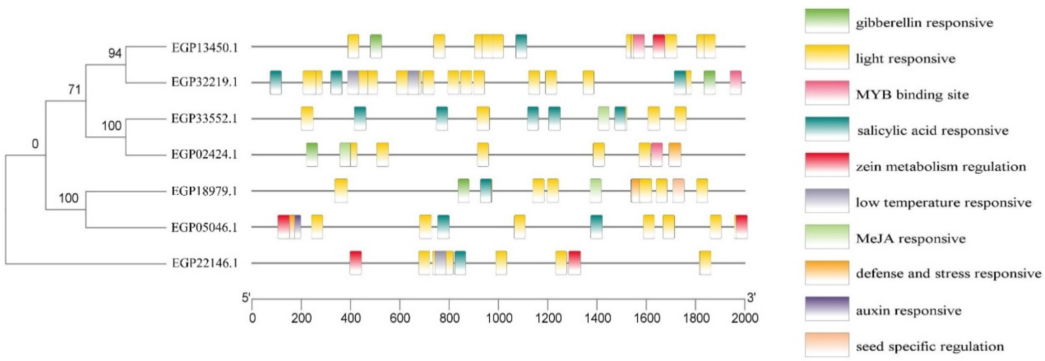

Supplementary Figure S2. Cis-acting elements of seven *YABBYs* gene promoters in eggplant.
