# Supplementary material for: SmYABBY1, a Light-Inducible Transcription Factor, Positively Regulates Anthocyanin Biosynthesis in Eggplant (Solanum melongena L.)
Source: Int J Mol Sci. 2026 May 13;27(10):4347. doi: 10.3390/ijms27104347 (PMC13207839; doi:10.3390/ijms27104347)
Supplement: Supplementary file 1 [file ijms-27-04347-s001.zip › supplementary tables.pdf]

Table. S1 Primer sequences used in the study

Gene	Forward primer (5'-3')	Reverse primer (5'-3')
For qRT-PCR		
SmActin	GTCGGAATGGGACAGAAGGATG	GTGCCTCAGTCAGGAGAACAGGGT
SmCHS	GGGAACAGTACTCCGGCTAGCC	AACACCTGAAATTGGGTCTGAACCA
SmDFR	GGCCATTGAGACTTGCCGACAG	CACCATTGGTCAACTGTCCTGTACT
SmANS	CTCGATTCCCACCTCGGACCTT	TCAGCTGCAGCGTCCTGTTTGT
AtActin	GTCTGGATTGGAGGGTC	TGAGAAATGGTCGGAAA
AtCHS	TGAGAACCATGTGCTTCAGG	CAGATGCATGTGACGTTTCC
AtCHI	TTTGTACCGTCCGTCAAGTC	CAATGACGGTGAAGATCACG
AtANS	TCAAGAAAGCCGGAGAAGAG	TTGTCCACTCGCGTTGTTAG
AtDFR	GTCGGTCCATTCATCACAAC	TGAGCGTTGCATAAGTCGTC
NtActin	AATGGAACTGGAATGGTCAAGGC	TGCCAGATCTTCTCCATGTCATCCCA
NtCHS	TTGTTCGAGCTTGTCTCTGC	AGCCCAGGAACATCTTTGAG
NtCHI	GTCAGGCCATTGAAAAGCTC	CTAATCGTCAATGCCCCAAC
NtF3H	CAAGGCATGTGTGGATATGG	TGTGTCGTTTCAGTCCAAGG
NtDFR	AACCAACAGTCAGGGGAATG	TTGGACATCGACAGTTCCAG
NtANS	TGGCGTTGAAGCTCATACTG	GGAATTAGGCACACACTTTGC
SmMYB75	GAGGTGACTTCGCTGCGGATG	TCACATCATTCGCTGTCCTTCCTG
SmYABBY1	TCCAAAGCCACCAGTCGTCA	ACTGAAGGCCTCCCTGTGAC
For Dual-LUC		
SmMYB75-YFP	CTCTCTCTCAAGCTTGGATCCATGAATAATCCTCCTATAATGTG	GCCCTTGCTCACCATACTAGTTTAATCAAGTAGATTCCACAAGTC
SmYABBY1-YFP	CTCTCTCTCAAGCTTGGATCCATGTCATCTTCATCTGCTCCGGACC	GCCCTTGCTCACCATACTAGTGTAAGGAGCTACACCAATGTTT
SmDFR-PG	CTTGATATCGAATTCCTGCAGACATGATTTACATCATAATTGTAG	GCTCTAGAACTAGTGGATCCATCTCAAAGTAACTGAACTTAGT
SmANS-PG	CTTGATATCGAATTCCTGCAGCACGTGGTGTTCAATAAC	GCTCTAGAACTAGTGGATCCCTCTTTAACGCGGAGTAC

SmMYB75-PG	CTTGATATCGAATTCCTGCAGCTAACACTAACATTATCTGTTTG	GCTCTAGAACTAGTGGATCC CACATTATAGGAGGATTATTCAT
For yeast one-hybrid		
SmYABBY1-42AD	TGCCTCTCCCGAATTCATGTCATCTTCATCTGCTCCGGACC	TCCAAAGCTTCTCGAG GTAAGGAGCTACACCAATGTTT
MYB75-Placzi	TATTGGATCGGAATTCTGCAAGTCTATCAAACAAGGAAG	GAGCACATGCCTCGAG CACATTATAGGAGGATTATTCATGA
SmDFR-Placzi	TATTGGATCGGAATTCACATGATTTACATCATAATTGTAG	GAGCACATGCCTCGAGATCTCAAAGTAACTGAACTTAGT
SmANS-Placzi	TATTGGATCGGAATTCCACGTGGTGTTCAATAAC	GAGCACATGCCTCGAGCTCTTTAACGCGGAGTAC
SmMYB75-Placzi	TATTGGATCGGAATTCCTAACACTAACATTATCTGTTTG	GAGCACATGCCTCGAG CACATTATAGGAGGATTATTCAT
For yeast two-hybrid		
SmMYB75-AD	GGAGGCCAGTGAATTCATGAATAATCCTCCTATAATGTG	CGAGCTCGATGGATCCTTAATCAAGTAGATTCCACAAGTC
SmYABBY1-BD	TCAGAGGAGGACCTGCATATGATGTCATCTTCATCTGCTCCGGACC	ATGCGGCCGCTGCAGGTCGACTAGTAAGGAGCTACACCAATGTTT
SmYABBY1-AD	GGAGGCCAGTGAATTCATGTCATCTTCATCTGCTCCGGACC	CGAGCTCGATGGATCCTAGTAAGGAGCTACACCAATGTTT
SmCOP1-BD	TCAGAGGAGGACCTGCATATGATG ATGGAAAGTTCCATTGGAGG	ATGCGGCCGCTGCAGGTCGAC GCTGCAAGGACCAACACTTTTA
For BiFC		
SmMYB75-106	GGACGCCGGCGGATCCATGAATAATCCTCCTATAATGTG	AGCTCTGCAGGTCGACATCAAGTAGATTCCACAAGTC
SmCOP1-104	AGGTACCCGGGGATCCATGATGGAAAGTTCCATTGGAGG	TGCCACCGCCGTCGACAGCTGCAAGGACCAACACTTTTA
SmCOP1-106	GGACGCCGGCGGATCCATGATGGAAAGTTCCATTGGAGG	AGCTGCAAGGACCAACACTTTTAAGCTGCAAGGACCAACACTTTTA
SmYABBY1-104	AGGTACCCGGGGATCCATGTCATCTTCATCTGCTCCGGACC	TGCCACCGCCGTCGACTTAGTAAGGAGCTACACCAATGTTT
Construction of vector universal primers		
PHB-YFP	GGAGAGGACACGCTGAAATCACCAG	TGTGGCCGTTTACGTCGC
42AD-R	ACCAGCCTCTTGCTGAGTGGAGAT	CGTAAATTTCTGGCAAGGTAGAC
Placzi	ATGCTGCCACTCCTCAATTGGAT	ATATACATACAGAGCACATGCC
T7	TAATACGACTCACTATAGGGCGA	
pXY106	GCGACACCCTGGTGAACCG	TCCATTTCACAGTTCGATAGCGA
